# A novel effect of bevacizumab in reducing characteristic pigmentation in Peutz–Jeghers syndrome: a case report and literature review

**DOI:** 10.3389/fonc.2025.1694187

**Published:** 2026-01-07

**Authors:** Delu Wu, Xinyue Liu, Song Wang, Huanyu Zhang, Guofeng Qu, Lina Wang

**Affiliations:** Department of Radiotherapy, The First Clinical Medical College of Lanzhou University, Lanzhou, Gansu, China

**Keywords:** Peutz-Jeghers syndrome, gastric-type endocervical adenocarcinoma, labial melanotic macules, bevacizumab, chemoradiation

## Abstract

Peutz–Jeghers syndrome (PJS) is a rare autosomal dominant disorder characterized by mucocutaneous pigmentation (e.g., perioral melanotic macules) and gastrointestinal hamartomatous polyps, with heightened cancer susceptibility. This report describes a 34-year-old Han Chinese ethnicity woman with familial Peutz–Jeghers syndrome (PJS) and stage IIA HPV-independent gastric-type endocervical adenocarcinoma following bevacizumab therapy. Initial concurrent chemoradiation (paclitaxel with either nedaplatin or cisplatin with volumetric modulated arc therapy) achieved partial response, while subsequent maintenance therapy combining bevacizumab with chemotherapy induced complete radiographic remission. Crucially, progressive fading of pathognomonic perioral melanotic macules demonstrated temporal correlation with bevacizumab administration, with sustained remission at 1-year follow-up. These findings challenge the conventional paradigm of PJS pigmentation as a static feature by demonstrating that VEGF-mediated angiogenesis concurrently drives carcinogenesis and melanin deposition. The case highlights three key implications, namely: (1) validation of bevacizumab’s dual therapeutic potential against PJS-associated malignancies and cutaneous manifestations, (2) proposal of mucocutaneous pigmentation as a dynamic biomarker for treatment response monitoring, and (3) urgent need for prospective clinical trials evaluating VEGF inhibition in PJS through longitudinal surveillance of oncologic control and pigment dynamics.

## Background

Peutz–Jeghers syndrome (PJS), a rare autosomal dominant genetic disorder, manifests pathognomonically through mucocutaneous pigmentation (particularly perioral melanotic macules) and the development of hamartomatous polyposis predominantly in the gastrointestinal tract. This condition carries significant oncogenic potential, with affected individuals demonstrating predisposition to various malignancies, particularly gastrointestinal carcinomas. We present a 34-year-old female proband with familial PJS who underwent multimodal therapy for cervical carcinoma. The patient initially received liposomal paclitaxel with nedaplatin as systemic chemotherapy, which was subsequently transitioned to a paclitaxel–cisplatin regimen administered concurrently with radiotherapy as part of definitive chemoradiation. This treatment approach achieved partial response, and the patient then began maintenance therapy combining bevacizumab with chemotherapy, which culminated in complete radiographic remission. Notably, the therapeutic intervention induced progressive attenuation of characteristic orofacial melanin deposition, with chronological correlation to bevacizumab administration. The treatment course exhibited favorable tolerability without significant adverse events. Mechanistically, bevacizumab—a recombinant humanized monoclonal antibody targeting vascular endothelial growth factor (VEGF)—mediates its anti-neoplastic effects through angiogenesis inhibition, a pathway increasingly recognized as potentially modifiable in PJS-related tumorigenesis. This observation suggests a dual therapeutic potential encompassing both neoplastic control and cutaneous manifestation amelioration in PJS patients.

## Case presentation

**Figure 1:**
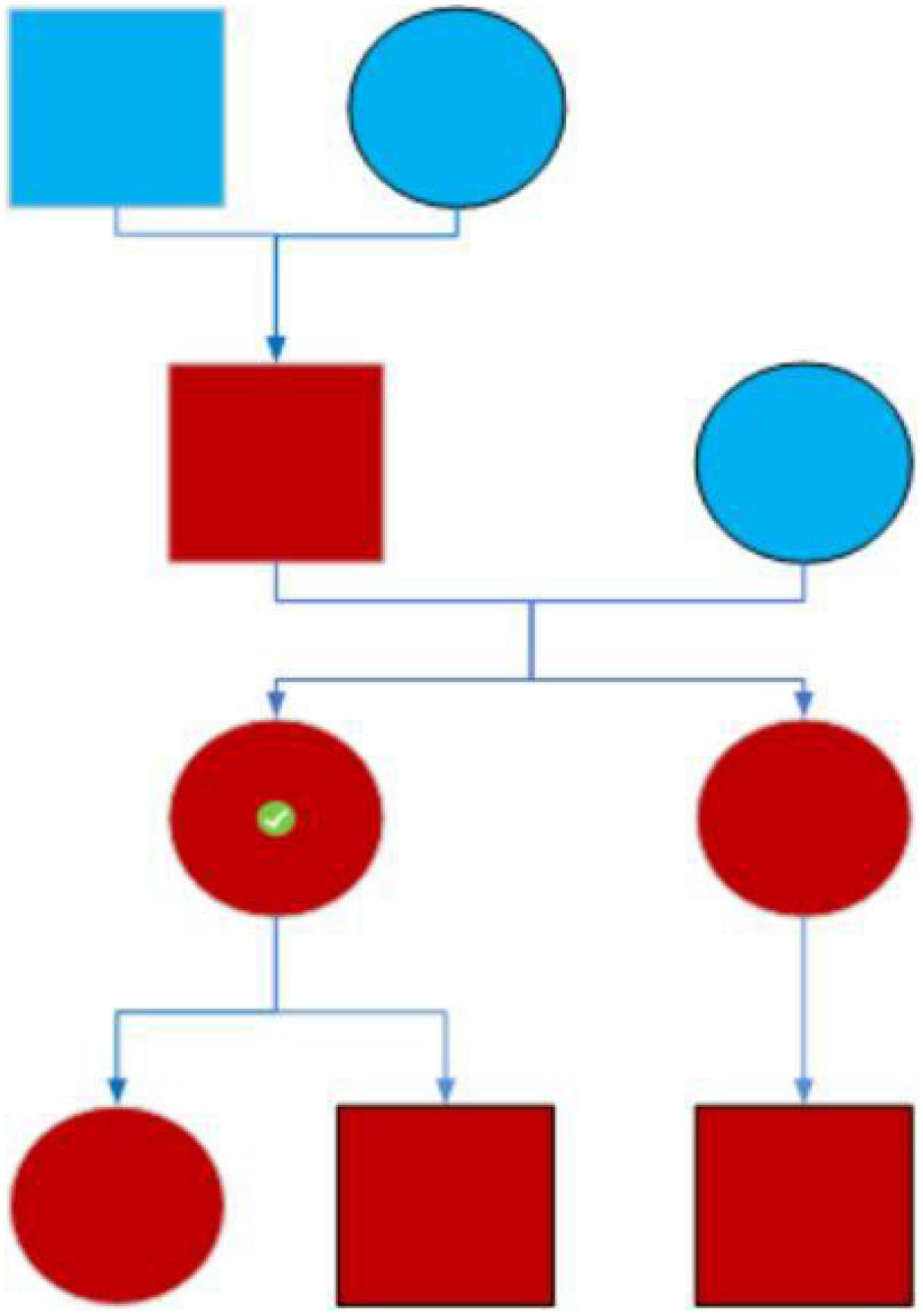
Pedigree chart of a family with Peutz–Jeghers syndrome. Symbol key: Red symbols: Individuals exhibiting mucocutaneous pigmentation. Circles: Female family members. Squares: Male family members. Symbol with checkmark (✓): Index case (proband).

A 34-year-old Han Chinese ethnicity female patient (Ms. Wu XX) was admitted to the Gastroenterology Department in June 2022 with a 6-month history of diarrhea and hematochezia. Her medical history included open abdominal surgery for intestinal obstruction at age 13. A physical examination revealed multiple perioral melanotic macules. The patient reported a family history of intestinal polyposis and perioral pigmentation in her father and children (see genetic pedigree in **Figure 1**). No other significant medical or familial history was noted.

Imaging and endoscopic findings (June 2022):

Thoracic and abdominal CT:

Multiple intraluminal enhancing nodules in the gastric body (greater curvature), duodenum, pelvic small bowel, and ascending colon.

Endometrial thickening with heterogeneous myometrial enhancement (suspected fibroids).

Endoscopy:

Gastroscopy: Multiple gastric and duodenal polyps.

Colonoscopy: Small bowel polyposis (consistent with Peutz–Jeghers syndrome).

Histopathology (gastric biopsy):

Hyperplastic polyps with focal mild epithelial dysplasia.

Fundic gland polyps.

Clinical course:

December 2022: Re-admitted for abdominal distension. Repeat CT showed progression of duodenal, small bowel, and colonic polyps. Underwent enteroscopic polypectomy.

March 2023: Evaluated for recurrent diarrhea and hematochezia:

Tumor markers: CA-125: 123.00 U/mL, CA19-9: 462.00 U/mL, CA72-4: 8.60 U/mL.

**Figure 2:**
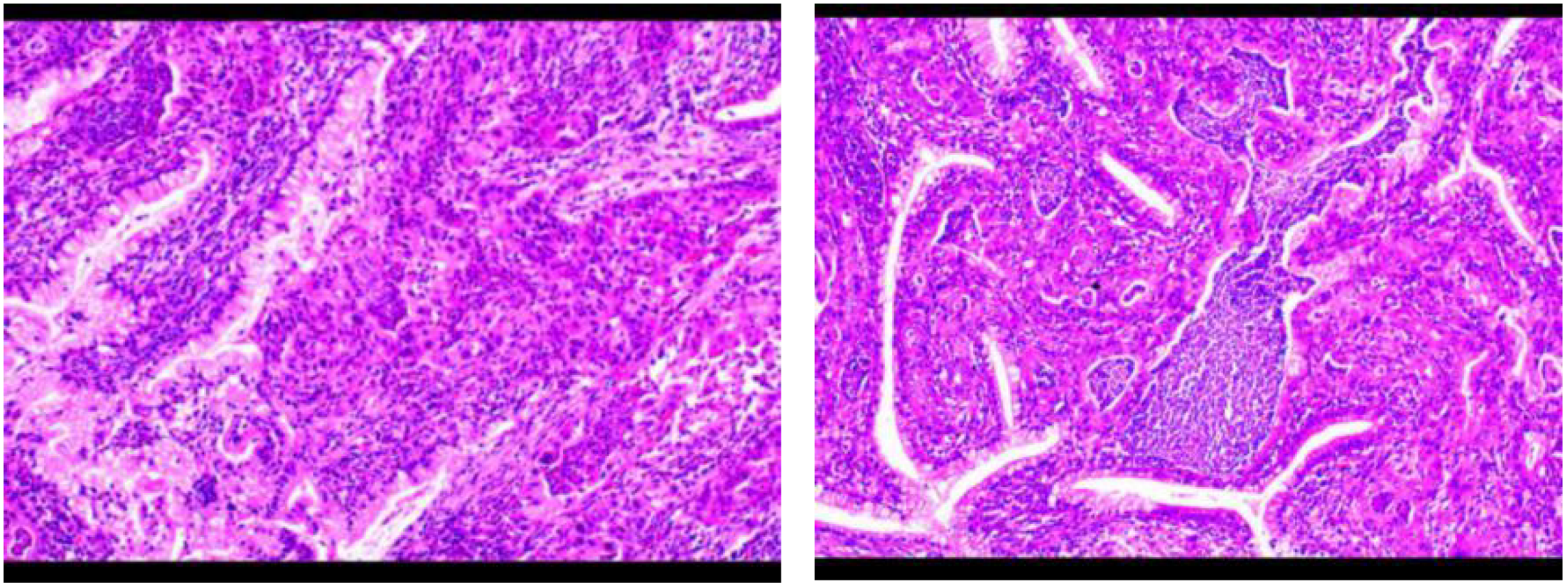
Histopathological findings of cervical cancer in a patient with Peutz–Jeghers syndrome. The tumor tissue exhibits irregular glandular and cribriform patterns, characterized by cells with large, hyperchromatic nuclei and abundant cytoplasm, demonstrating invasive growth.

Transvaginal ultrasound (**Figure 2**): Cervical mass, endometrial thickening, and uterine fibroids.

Cervical biopsy: HPV-independent gastric-type endocervical adenocarcinoma (moderately differentiated).

Immunohistochemistry: ER (-), PR (-), PTEN (weak+), PAX-8 (focal+), Ki-67 (50% in hotspot areas), p53 (mutant pattern), CK7 (+), CK20 (-), p16 (patchy+).

Imaging staging:

PET/CT:

Hypermetabolic cervical lesion (right posterior wall) involving the posterior vaginal fornix (FIGO IIA).

Mild FDG uptake in duodenal, ileocecal, and colorectal polyps (consistent with PJS).

Post-polypectomy changes without residual hypermetabolism.

Pelvic MRI: Cervical cancer (T2b), Nabothian cysts, posterior uterine fibroid, and sigmoid polyps.

Endoscopic interventions (2023):

Gastroscopy: Numerous duodenal polyps (2.0–5.0 cm), with 5 large proximal polyps resected.

Colonoscopy: 12 polyps (>2 cm) removed from cecum to sigmoid colon.

Oncologic treatment:

April–May 2023: Received liposomal paclitaxel 200 mg + nedaplatin 120 mg (2 cycles).

June 2023: Volumetric modulated arc therapy (VMAT):

PTV50.4: 5040 cGy/28 fractions.

PGTVnd: 6020 cGy/28 fractions.

Intracavitary brachytherapy: HR-CTV 3000 cGy/5 fractions.

Concurrent chemotherapy (TP regimen):

Nab-paclitaxel 400 mg (d1) + cisplatin 30 mg (d1-3).

September 2023: Partial response (PR) on MRI.

October 2023: TP regimen + bevacizumab 500 mg (d1).

November 2023: Stable disease on MRI.

Follow-up (1 year):

Achieved complete response (CR) with no cervical recurrence.

**Figure 3:**
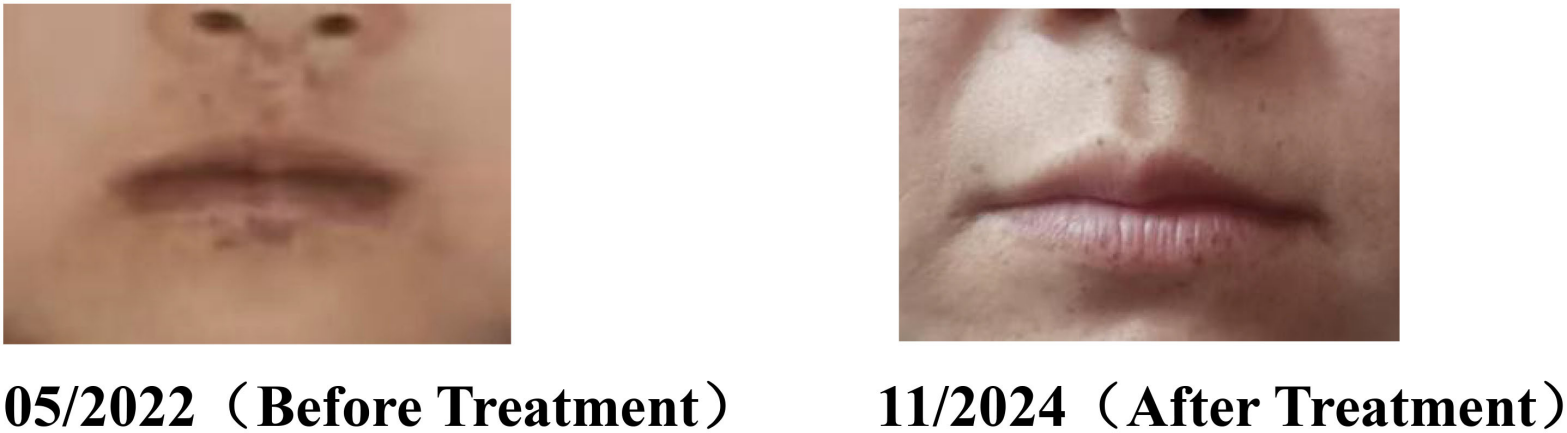
Comparative analysis of perioral melanotic macules in a Peutz–Jeghers syndrome patient before and after treatment.

Notable lightening of perioral pigmentation following polypectomy and bevacizumab therapy (**Figure 3**).

**Figure 4:**
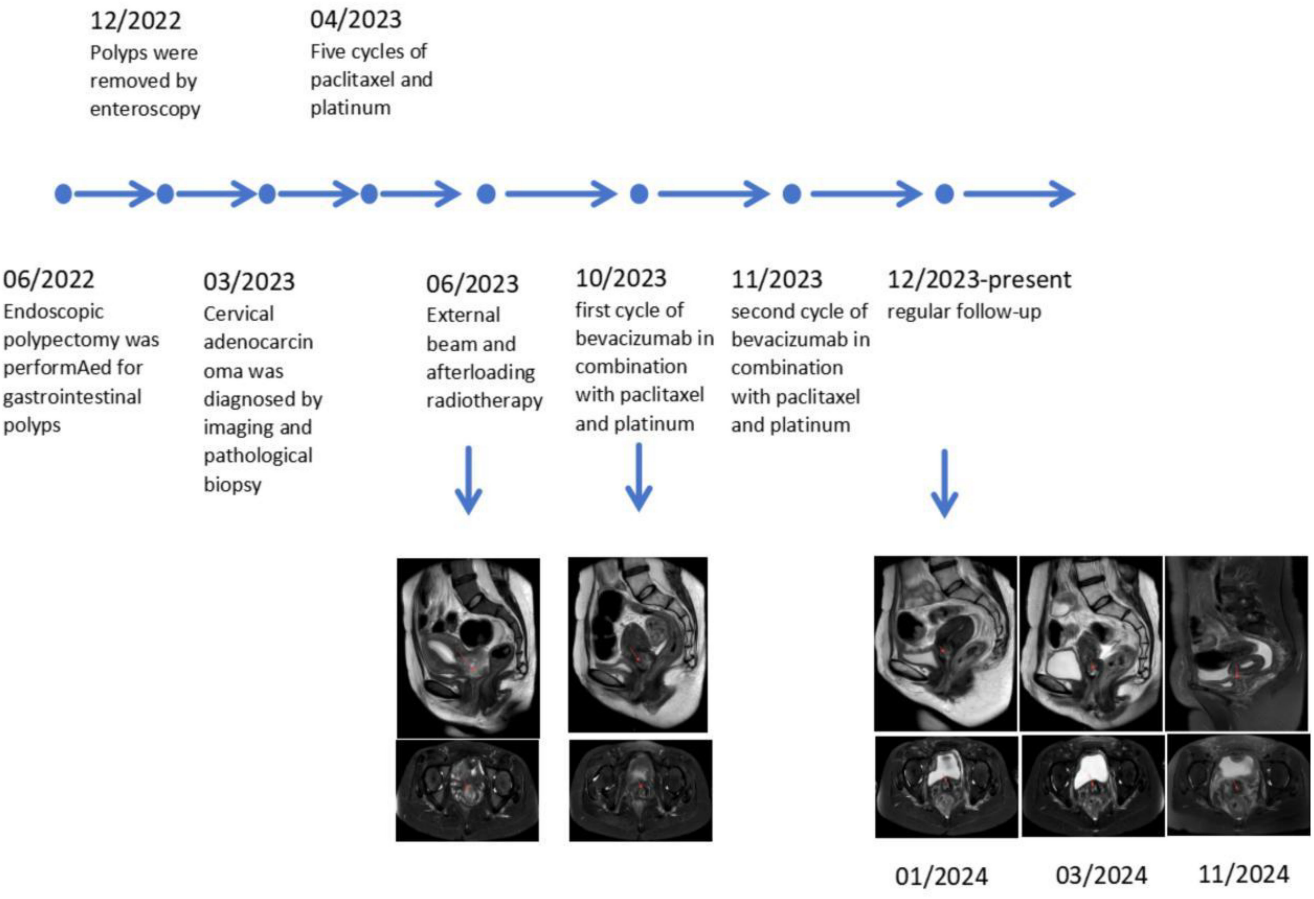
Therapeutic timeline and radiographic evolution in a Peutz–Jeghers syndrome patient.

Full therapeutic timeline documented (**Figure 4**).

Unaffected individuals: Non-consanguineous family members showed no phenotypic abnormalities.

Diagnostic considerations:

The diagnosis of Peutz–Jeghers syndrome (PJS) was established based on clinical criteria, as genetic testing was not performed due to financial constraints. According to established diagnostic guidelines (1), PJS can be confirmed by meeting any of the following criteria:

1. Presence of two or more histologically confirmed Peutz–Jeghers-type hamartomatous polyps.
2. Any Peutz–Jeghers polyp in conjunction with a family history of PJS.
3. Characteristic mucocutaneous pigmentation (involving oral mucosa, lips, nasal mucosa, periorbital region, genitalia, or digits) with a positive family history of PJS.
4. Characteristic mucocutaneous pigmentation and any Peutz–Jeghers polyp.

## Discussion

Peutz–Jeghers syndrome (PJS) is an autosomal dominant disorder with an estimated incidence ranging from 1:200,000 to 1:50,000 (2). The primary pathogenic mechanism involves loss-of-function mutations in the STK11/LKB1 gene, which disrupts multiple signaling pathways, including AMPK-mTOR, Wnt/β-catenin, and PI3K/AKT, leading to polyp formation and increased cancer susceptibility (1, 3, 4). STK11/LKB1 acts as a central regulator of cellular metabolism and proliferation. Its inactivation impairs AMPK-mediated inhibition of mTORC1, resulting in metabolic reprogramming characterized by enhanced glycolysis and lipid synthesis (the “Warburg effect”) to fuel tumor growth (5). Furthermore, STK11/LKB1 deficiency dysregulates the Hippo pathway by promoting nuclear translocation of YAP/TAZ, thereby activating pro-proliferative genes (e.g., CTGF, CYR61) and accelerating gastrointestinal polypogenesis (6). STK11/LKB1 loss also remodels the tumor microenvironment through PI3K/AKT–FOXO axis-mediated apoptosis suppression and aberrant activation of Wnt/β-catenin and Notch pathways, driving epithelial–mesenchymal transition (EMT) and cancer stemness (7, 8). Additionally, disruption of the LKB1–STRAD–MO25 complex compromises intestinal epithelial polarity via PAR1/MARK dysfunction, further impairing barrier integrity (9). Emerging evidence suggests that STK11/LKB1 modulates mitochondrial autophagy and ROS metabolism, forming a dynamic feedback loop with the p53–BAX apoptotic pathway (10, 11), which may explain the early-onset and multifocal malignancies in PJS. Collectively, these mechanisms reflect a triad of metabolic, polarity, and epigenetic dysregulation. Therapeutic strategies targeting the AMPK/mTOR–Hippo axis or combining epigenetic modifiers (e.g., HDAC inhibitors) may offer novel intervention opportunities (12).

Endoscopic polypectomy remains the cornerstone for managing gastrointestinal polyps in PJS. Balloon-assisted enteroscopy (BAE) has significantly reduced surgical needs, enabling the safe resection of polyps ≥1 cm to mitigate intussusception and obstruction risks, though repeated procedures are often required for complete small bowel surveillance (13, 14). Gastroscopy and colonoscopy effectively monitor and remove gastric/colorectal polyps with low complication rates (e.g., 0.25% perforation risk) (14). Surgery is reserved for complications (e.g., bowel necrosis, irreducible intussusception) or malignancies but may increase adhesion-related morbidity (13, 15).

Conventional approaches fail to prevent polyp recurrence or malignant transformation. PJS patients face a 37%–93% lifetime cancer risk, notably colorectal, breast, small intestinal, gastric, and pancreatic cancers (16). Recent studies focus on molecular mechanisms and targeted therapies for PJS-associated malignancies (**Table 1**). Cervical gastric-type adenocarcinoma (G-EAC), occurring in 11% of female PJS patients (17), exhibits aggressive behavior with early metastasis and poor response to conventional screening (TCT/HPV testing) (18). Treatment relies on surgery combined with chemoradiation and targeted therapies (**Table 2**), though outcomes remain inferior to HPV-related cervical cancer (19, 20). Preclinical studies explore mTOR inhibitors (e.g., everolimus), PARP inhibitors (e.g., olaparib), and AXL inhibitors (e.g., bemcentinib) to address STK11-related metabolic and DNA repair defects. Notably, combining bemcentinib with PD-1 inhibitors may overcome immunotherapy resistance in STK11-mutant tumors (20). Genetic counseling and preimplantation genetic testing (PGT) effectively prevent STK11 mutation transmission, with successful clinical applications reported (21). For mucocutaneous pigmentation, picosecond laser therapy shows promising cosmetic outcomes (22, 23).

**Table 1:** Pathogenic pathways and therapeutic targets in Peutz–Jeghers syndrome.

Pathogenic pathway	Mechanistic basis	Potential targets/drugs	References
AMPK/mTOR pathway	STK11/LKB1 inactivation reduces AMPK activity, leading to mTOR hyperactivation and tumorigenesis	AMPK activators (metformin), mTOR inhibitors (rapamycin)	(5, 24)
Cell polarity regulation	LKB1 loss disrupts PAR-4/MARK kinase-mediated polarity, inducing structural chaos and carcinogenesis	PAR-4/MARK pathway modulators	(25–27)
HIF-1α pathway	LKB1 deficiency stabilizes HIF-1α, enhancing angiogenesis and tumor microenvironment adaptation	HIF-1α inhibitors (bevacizumab)	(28–30)
MAPK/KRAS pathway	Co-activation of KRAS and MAPK signaling driven by STK11/LKB1 mutations	MEK inhibitors (trametinib)	(31, 32)
Wnt/β-catenin pathway	Aberrant Wnt activation with β-catenin accumulation in polyps due to LKB1 deficiency	Wnt inhibitors (PORCN inhibitors)	(3, 33)

**Table 2:** Case reports of Peutz–Jeghers syndrome with gastric-type adenocarcinoma of the cervix (GAS).

Case no.	Age	PJS features (polyps/pigmentation)	GAS diagnostic methods	FIGO stage (2018)	Treatment modalities	Outcome	Reference	Publication year
1	32	Intestinal polyps, perioral pigmentation	Cervical biopsy + MRI	IB3	NACT + RH + ACT	Unknown	(34)	2023
2	39	Intestinal polyps, perioral/periocular/acral pigmentation	Cervical biopsy + MRI	IB3	RH + CCRT	NED at 1-year follow-up	(35)	2021
3	32	Unknown	Abdominal CT + IHC	IV	RH + TP + bevacizumab	Died at 1 year (multiple metastases)	(36)	2023
4	31	Intestinal polyps, perioral/acral pigmentation	Pelvic MRI + cone biopsy + IHC	IB2	RH + paclitaxel/oxaliplatin (4 cycles)	NED at 5-year follow-up	(17)	2023
5	45	Gastrointestinal polyps, perioral/periocular pigmentation	Pelvic MRI + histopathology	IIIC	RH + docetaxel/carboplatin + bevacizumab	CR, NED at 15-month follow-up	(37)	2021
6	36	Small bowel polyps	Pelvic MRI + cone biopsy + histopathology	IB2	RH + CCRT (details unknown)	Unknown	(38)	2019
7	24	Intestinal polyps, perioral pigmentation	Pelvic MRI + IHC	IIIA	RH + adjuvant RT	NED at 12-month follow-up	(39)	2019
8	24	Intestinal polyps, lip/buccal mucosa/acral pigmentation	Pelvic MRI + IHC	Unknown	Unknown	Unknown	(40)	2024
9	32	Small bowel polyps, lip/digital mucocutaneous pigmentation	Pelvic MRI + IHC	Unknown	Total hysterectomy	Unknown	(41)	2015
10	38	Unknown	Pelvic MRI + histopathology	Unknown	RH + carboplatin (6 cycles)	NED at 18-month follow-up	(42)	2024

The cervical adenocarcinoma in this case exhibited characteristic features of gastric-type adenocarcinoma (GAS), including HPV negativity and a distinctive immunohistochemical profile: ER (-), PR (-), PTEN (weak+), p53 (mutant pattern), CDX-2 (-), SATB2 (-), villin (-), CK7 (+), CK20 (-), VIM (-), and p16 (patchy+). These findings are consistent with PJS-associated cervical cancer. For limited-stage cervical cancer (IIA2), concurrent chemoradiation (IMRT + cisplatin) remains the standard treatment. However, for GAS-type cervical cancer, the TP regimen (paclitaxel + platinum) is often preferred. Bevacizumab, a monoclonal antibody targeting VEGF, inhibits tumor angiogenesis and is approved for cervical and colorectal cancers. In this case, the addition of bevacizumab to the TP regimen resulted in tumor regression and a complete response (CR), suggesting its potential efficacy in PJS-associated cervical cancer.

The mucocutaneous pigmentation in PJS is primarily caused by localized melanin deposition and dermal vascular proliferation, linked to AMPK/mTOR signaling dysregulation due to STK11/LKB1 inactivation. STK11 mutations activate the mTOR pathway, upregulating HIF-1α and VEGF expression, which stimulates angiogenesis and melanocyte activity (43, 44). VEGF not only promotes tumor angiogenesis but also induces melanocyte proliferation and migration through paracrine signaling (45). Thus, the pigmentation may reflect the systemic activation of the VEGF/mTOR pathway rather than being merely a cutaneous manifestation. Bevacizumab, by inhibiting VEGF and its downstream signaling (e.g., PI3K/AKT/mTOR) (46), may reduce melanocyte activity and melanin deposition, leading to pigmentation regression. Although this phenomenon has not been widely reported in PJS patients, this case suggests that VEGF signaling may play a significant role in the pathogenesis of mucocutaneous pigmentation in PJS. Furthermore, whether pigmentation changes can serve as a biomarker for treatment response in PJS warrants further investigation.

## Future perspectives and therapeutic potential

Our study reveals that the VEGF/PI3K/AKT/mTOR signaling axis may concurrently regulate both carcinogenesis and melanin deposition in the pathogenesis of PJS, opening promising avenues to develop novel therapeutic strategies. Beyond conventional synthetic inhibitors, several natural compounds have demonstrated efficacy in modulating these key signaling pathways and warrant further investigation. Notable candidates include conjugated linoleic acid, recognized for its health-promoting properties, and epigallocatechin-3-gallate—the predominant polyphenol in green tea—which not only effectively suppresses PI3K/AKT/mTOR signaling and VEGF expression but also exhibits synergistic effects with chemotherapeutic agents in preclinical studies (47). Although we propose a mechanism whereby bevacizumab may ameliorate cutaneous hyperpigmentation by inhibiting VEGF-driven melanocyte activity—a hypothesis physiologically plausible and supported by our clinical observations—it must be emphasized that this association remains correlative and requires further validation. Our study currently lacks direct histopathological evidence, including comparative analyses of melanin content, melanocyte density, and CD31-stained microvessel density in lesions before and after treatment as well as biochemical quantification of local VEGF levels. Therefore, the inferred causal relationship should be interpreted with caution. Building on these findings, future research should establish STK11-deficient models to systematically evaluate the efficacy of natural compounds in reducing mucocutaneous pigmentation and suppressing hamartomatous polyp growth while simultaneously addressing the evidence gaps in our current study. Specifically, pre- and post-intervention histopathological analyses of model lesions—quantifying changes in melanin content, melanocyte density, microvessel density, and local VEGF levels—would provide direct evidence for the proposed mechanism. Further clinical investigations could explore the potential of natural compounds as adjuncts to targeted therapies such as bevacizumab, employing multidimensional assessments to validate synergistic effects. This approach aims to enhance therapeutic efficacy, reduce dosage requirements, minimize adverse effects, and ultimately improve long-term outcomes for PJS patients.

## Author’s note

Bevacizumab is a registered trademark of Qilu Pharmaceutical Co., LTD; however, no financial or promotional relationships exist between the authors and any commercial entity associated with this drug.

## Data Availability

The original contributions presented in the study are included in the article/Supplementary Material. Further inquiries can be directed to the corresponding author.

## References

[1] Beggs AD Latchford AR Vasen HF Moslein G Alonso A Aretz S . Peutz-Jeghers syndrome: a systematic review and recommendations for management. Gut. (2010) 59:975–86. doi: 10.1136/gut.2009.198499, PMID: 20581245

[2] Giardiello FM Trimbath JD . Peutz-Jeghers syndrome and management recommendations. Clin Gastroenterol Hepatol. (2006) 4:408–15. doi: 10.1016/j.cgh.2005.11.005, PMID: 16616343

[3] Hsieh MJ Weng CC Lin YC Wu CC Chen LT Cheng KH . Inhibition of β-catenin activity abolishes LKB1 loss-driven pancreatic cystadenoma in mice. Int J Mol Sci. (2021) 22:4649. doi: 10.3390/ijms22094649, PMID: 33924999 PMC8125161

[4] Glaviano A Foo ASC Lam HY Yap KCH Jacot W Jones RH . PI3K/AKT/mTOR signaling transduction pathway and targeted therapies in cancer. Mol Cancer. (2023) 22:138. doi: 10.1186/s12943-023-01827-6, PMID: 37596643 PMC10436543

[5] Shackelford DB Shaw RJ . The LKB1-AMPK pathway: metabolism and growth control in tumour suppression. Nat Rev Cancer. (2009) 9:563–75. doi: 10.1038/nrc2676, PMID: 19629071 PMC2756045

[6] Mohseni M Sun J Lau A Curtis S Goldsmith J Fox VL . A genetic screen identifies an LKB1-MARK signalling axis controlling the Hippo-YAP pathway. Nat Cell Biol. (2014) 16:108–17. doi: 10.1038/ncb2884, PMID: 24362629 PMC4159053

[7] Ji H Ramsey MR Hayes DN Fan C McNamara K Kozlowski P . LKB1 modulates lung cancer differentiation and metastasis. Nature. (2007) 448:807–10. doi: 10.1038/nature06030, PMID: 17676035

[8] Ollila S Mäkelä TP . The tumor suppressor kinase LKB1: lessons from mouse models. J Mol Cell Biol. (2011) 3:330–40. doi: 10.1093/jmcb/mjr016, PMID: 21926085

[9] Xia T Chen D Liu X Qi H Wang W Chen H . Midkine noncanonically suppresses AMPK activation through disrupting the LKB1-STRAD-Mo25 complex. Cell Death Dis. (2022) 13:414. doi: 10.1038/s41419-022-04801-0, PMID: 35487917 PMC9054788

[10] Choi EJ Oh HT Lee SH Zhang CS Li M Kim SY . Metabolic stress induces a double-positive feedback loop between AMPK and SQSTM1/p62 conferring dual activation of AMPK and NFE2L2/NRF2 to synergize antioxidant defense. Autophagy. (2024) 20:2490–510. doi: 10.1080/15548627.2024.2374692, PMID: 38953310 PMC11572134

[11] Feng Y Chen Y Wu X Chen J Zhou Q Liu B . Interplay of energy metabolism and autophagy. Autophagy. (2024) 20:4–14. doi: 10.1080/15548627.2023.2247300, PMID: 37594406 PMC10761056

[12] Cheng B Pan W Xiao Y Ding Z Zhou Y Fei X . HDAC-targeting epigenetic modulators for cancer immunotherapy. Eur J Med Chem. (2024) 265:116129. doi: 10.1016/j.ejmech.2024.116129, PMID: 38211468

[13] Dai YC Xiao B Zhang YL Zhi FC Jiang B Zhou DY . Endoscopic and Surgical Treatment of 52 Cases of Peutz-Jeghers Syndrome. Journal of Clinical Internal Medicine. (2005) 22:455–7. doi: 10.3969/j.issn.1001-9057.2005.07.009

[14] Zhu HY Du YQ . Progress in Small Bowel Endoscopy Treatment for Peutz-Jeghers Syndrome. Journal of Colorectal & Anal Surgery. (2023) 29:122–6. doi: 10.19668/j.cnki.issn1674-0491.2023.02.004

[15] Luo MW Zhang ZQ Wei Y Yan WD Yan DW . A Case Report of Peutz-Jeghers Syndrome Complicated with Jejunal Intussusception Obstruction. Journal of Shanghai Jiaotong University (Medical Science). (2021) 41:1129–32. doi: 10.3969/j.issn.1674-8115.2021.08.023

[16] van Lier MG Wagner A Mathus-Vliegen EM Kuipers EJ Steyerberg EW van Leerdam ME . High cancer risk in Peutz-Jeghers syndrome: a systematic review and surveillance recommendations. Am J Gastroenterol. (2010) 105:1258–64. doi: 10.1038/ajg.2009.725, PMID: 20051941

[17] Li X Qi Y Zhang W Rao Y Zhang N Qu P . Peutz-Jeghers syndrome with gastric-type mucinous endocervical adenocarcinoma and sex-cord tumor with annular tubules: A case report. Front Med (Lausanne). (2023) 10:1094839. doi: 10.3389/fmed.2023.1094839, PMID: 37025955 PMC10072263

[18] Karamurzin YS Kiyokawa T Parkash V Jotwani AR Patel P Pike MC . Gastric-type endocervical adenocarcinoma: an aggressive tumor with unusual metastatic patterns and poor prognosis. Am J Surg Pathol. (2015) 39:1449–57. doi: 10.1097/PAS.0000000000000532, PMID: 26457350 PMC4976691

[19] Wang WL Ye H . Research Progress on Peutz-Jeghers Syndrome-Associated Gastric-Type Adenocarcinoma of the Cervix. Chinese Journal of Minimally Invasive Surgery. (2022) 2:167–9. doi: 10.3969/j.issn.1009-6604.2022.02.014

[20] Jiang AQ Kang Y Xu CJ . Research Progress on Peutz-Jeghers Syndrome-Associated Gastric-Type Adenocarcinoma of the Cervix. Chinese Journal of Practical Gynecology and Obstetrics. (2023) 39:1144–8. doi: 10.19538/j.fk2023110118

[21] Byrjalsen A Roos L Diemer T Karstensen JG Løssl K Jelsig AM . Preimplantation genetic testing in two Danish couples affected by Peutz-Jeghers syndrome. Scand J Gastroenterol. (2023) 58:314–8. doi: 10.1080/00365521.2022.2129031, PMID: 36200740

[22] Zeng R Wu Q Guo L Lin T . Successful treatment of oral pigmented spots in Chinese subjects with Peutz-Jeghers syndrome using a 755-nm picosecond laser. Indian J Dermatol Venereol Leprol. (2021) 87:288–9. doi: 10.25259/IJDVL_151_20, PMID: 33769737

[23] Agud-Dios M Perandones-Gonzalez H Fernández-Domper L Dominguez Santas M Boixeda de Miquel P . Picosecond 755-nm alexandrite laser treatment for oral lentiginosis in Peutz-Jeghers disease. Lasers Surg Med. (2022) 54:823–4. doi: 10.1002/lsm.23553, PMID: 35485783

[24] Gwinn DM Shackelford DB Egan DF Mihaylova MM Mery A Vasquez DS . AMPK phosphorylation of raptor mediates a metabolic checkpoint. Mol Cell. (2008) 30:214–26. doi: 10.1016/j.molcel.2008.03.003, PMID: 18439900 PMC2674027

[25] Lo B Strasser G Sagolla M Austin CD Junttila M Mellman I . Lkb1 regulates organogenesis and early oncogenesis along AMPK-dependent and -independent pathways. J Cell Biol. (2012) 199:1117–30. doi: 10.1083/jcb.201208080, PMID: 23266956 PMC3529533

[26] Partanen JI Tervonen TA Klefström J . Breaking the epithelial polarity barrier in cancer: the strange case of LKB1/PAR-4. Philos Trans R Soc Lond B Biol Sci. (2013) 368:20130111. doi: 10.1098/rstb.2013.0111, PMID: 24062587 PMC3785967

[27] Chan KT Asokan SB King SJ Bo T Dubose ES Liu W . LKB1 loss in melanoma disrupts directional migration toward extracellular matrix cues. J Cell Biol. (2014) 207:299–315. doi: 10.1083/jcb.201404067, PMID: 25349262 PMC4210439

[28] Ylikorkala A Rossi DJ Korsisaari N Luukko K Alitalo K Henkemeyer M . Vascular abnormalities and deregulation of VEGF in Lkb1-deficient mice. Science. (2001) 293:1323–6. doi: 10.1126/science.1062074, PMID: 11509733

[29] Brugarolas J Kaelin WG Jr . Dysregulation of HIF and VEGF is a unifying feature of the familial hamartoma syndromes. Cancer Cell. (2004) 6:7–10. doi: 10.1016/j.ccr.2004.06.020, PMID: 15261137

[30] Faubert B Vincent EE Griss T Samborska B Izreig S Svensson RU . Loss of the tumor suppressor LKB1 promotes metabolic reprogramming of cancer cells via HIF-1α. Proc Natl Acad Sci U S A. (2014) 111:2554–9. doi: 10.1073/pnas.1312570111, PMID: 24550282 PMC3932920

[31] Della Corte CM Ciaramella V Di Mauro C Castellone MD Papaccio F Fasano M . Metformin increases antitumor activity of MEK inhibitors through GLI1 downregulation in LKB1 positive human NSCLC cancer cells. Oncotarget. (2016) 7:4265–78. doi: 10.18632/oncotarget.6559, PMID: 26673006 PMC4826204

[32] Zinatizadeh MR Miri SR Zarandi PK Chalbatani GM Rapôso C Mirzaei HR . The Hippo Tumor Suppressor Pathway (YAP/TAZ/TEAD/MST/LATS) and EGFR-RAS-RAF-MEK in cancer metastasis. Genes Dis. (2021) 8:48–60. doi: 10.1016/j.gendis.2019.11.003, PMID: 33569513 PMC7859453

[33] Wang Y Liu J Zheng S Cao L Li Y Sheng R . The deubiquitinase USP10 mediates crosstalk between the LKB1/AMPK axis and Wnt/β-catenin signaling in cancer. FEBS Lett. (2023) 597:3061–71. doi: 10.1002/1873-3468.14763, PMID: 37873736

[34] Tong T Fan Q Shi S Li Y Wang Y . Gastric-type mucinous adenocarcinoma of the cervix in a woman with Peutz-Jeghers syndrome: a case report. Acta Chir Belg. (2023) 123:448–53. doi: 10.1080/00015458.2022.2040110, PMID: 35135434

[35] Vu Dinh G Doan Thi Hong N Vo Ngoc T Nguyen Thanh L Hoang Thi H Phung Thi H . Peuzt - Jeghers syndrome with gastric type mucinous endocervical adenocarcinoma in a young woman: A case report. Ann Med Surg (Lond). (2021) 69:102700. doi: 10.1016/j.amsu.2021.102700, PMID: 34429956 PMC8371195

[36] Wu X Liang D He Y . Peutz Jeghers syndrome accompanied with cervical gastric adenocarcinoma and extensive metastasis: a case report. Int J Clin Exp Pathol. (2023) 16:386–92., PMID: 38188351 PMC10767481

[37] Kotaka S Kanno K Yanai S Omori M Andou M . Peutz-Jeghers syndrome complicated by gastric-type cervical mucinous carcinoma and primary peritoneal carcinoma. J Obstet Gynaecol Res. (2021) 47:3732–6. doi: 10.1111/jog.14944, PMID: 34278661

[38] Kim Y Kim EY Kim TJ Lim KT Lee KH Chun Y . A rare case of gastric-type mucinous adenocarcinoma in a woman with Peutz-Jeghers syndrome. Obstet Gynecol Sci. (2019) 62:474–7. doi: 10.5468/ogs.2019.62.6.474, PMID: 31777745 PMC6856476

[39] Neyaz A Husain N Deodhar M Khurana R Shukla S Arora A . Synchronous cervical minimal deviation adenocarcinoma, gastric type adenocarcinoma and lobular endocervical glandular hyperplasia along with STIL in peutz-jeghers syndrome: eliciting oncogenesis pathways. Turk Patoloji Derg. (2019) 35:247–53. doi: 10.5146/tjpath.2017.01406, PMID: 28832082

[40] Yang L Duan D Xiong Y Liu T Zhao L Lai F . Preoperative multimodal ultrasonic imaging in a case of Peutz-Jeghers syndrome complicated by atypical lobular endocervical glandular hyperplasia: a case report and literature review. Hered Cancer Clin Pract. (2024) 22:3. doi: 10.1186/s13053-024-00275-7, PMID: 38419118 PMC10900695

[41] Peng WX Kure S Ishino K Kurose K Yoneyama K Wada R . P16-positive continuous minimal deviation adenocarcinoma and gastric type adenocarcinoma in a patient with Peutz-Jeghers syndrome. Int J Clin Exp Pathol. (2015) 8:5877–82., PMID: 26191312 PMC4503183

[42] Zhou Y Wang X Li Y Zhang W Xu X Pang Y . When synchronous mucinous metaplasia and neoplasia of the female genital tract and peutz-jeghers syndrome meet: a case report and literature reviews. BMC Womens Health. (2024) 24:375. doi: 10.1186/s12905-024-03184-y, PMID: 38937781 PMC11212150

[43] Amin RM Hiroshima K Miyagi Y Kokubo T Hoshi K Fujisawa T . Role of the PI3K/Akt, mTOR, and STK11/LKB1 pathways in the tumorigenesis of sclerosing hemangioma of the lung. Pathol Int. (2008) 58:38–44. doi: 10.1111/j.1440-1827.2007.02186.x, PMID: 18067639

[44] Shackelford DB Vasquez DS Corbeil J Wu S Leblanc M Wu CL . mTOR and HIF-1alpha-mediated tumor metabolism in an LKB1 mouse model of Peutz-Jeghers syndrome. Proc Natl Acad Sci U S A. (2009) 106:11137–42. doi: 10.1073/pnas.0900465106, PMID: 19541609 PMC2708689

[45] Parikh R Parikh S Berzin D Vaknine H Ovadia S Likonen D . Recycled melanoma-secreted melanosomes regulate tumor-associated macrophage diversification. EMBO J. (2024) 43:3553–86. doi: 10.1038/s44318-024-00103-7, PMID: 38719996 PMC11377571

[46] Rho SB Byun HJ Kim BR Lee CH . LKB1/STK11 tumor suppressor reduces angiogenesis by directly interacting with VEGFR2 in tumorigenesis. Biomol Ther (Seoul). (2023) 31:456–65. doi: 10.4062/biomolther.2023.106, PMID: 37357018 PMC10315345

[47] Javedan G Shidfar F Davoodi SH Ajami M Gorjipour F Sureda A . Conjugated linoleic acid rat pretreatment reduces renal damage in ischemia/reperfusion injury: Unraveling antiapoptotic mechanisms and regulation of phosphorylated mammalian target of rapamycin. Mol Nutr Food Res. (2016) 60:2665–77. doi: 10.1002/mnfr.201600112, PMID: 27466783

